# Catastrophic Antiphospholipid Syndrome: A Complex Diagnosis in the Setting of Lupus

**DOI:** 10.7759/cureus.42922

**Published:** 2023-08-03

**Authors:** Jessica Liang, Raai Mahmood, Ilyes Benchaala, Russel York, Housam Sarakbi

**Affiliations:** 1 Internal Medicine, Wayne State University Detroit Medical Center, Detroit, USA; 2 Rheumatology, Wayne State University Detroit Medical Center, Detroit, USA

**Keywords:** lupus mesenteric vasculitis, raynaud’s phenomenon, intravenous immunoglobulins (ivig), rituximab therapy, systemic lupus erythema, sle and lupus nephritis, antiphospholipid antibody syndrome (aps), catastrophic antiphospholipid syndrome (caps)

## Abstract

This case report aims to highlight the importance of keeping catastrophic antiphospholipid syndrome (CAPS) high on the list of differentials in patients with lupus who present with digital ischemia and to understand the workup and treatment of the disease. Catastrophic antiphospholipid syndrome is a life-threatening variant of antiphospholipid syndrome (APS), and it is distinguished on the APS spectrum by its increased intensity and extent of thrombotic outcomes. Less than 1% of patients with APS develop CAPS and the demographic of patients affected are primarily females, 37 ± 14 years old, and have underlying primary APS or systemic lupus erythematosus (SLE).

This is the case of a young female with lupus and end-stage renal disease secondary to lupus nephritis who presented to the emergency department for shortness of breath and bilateral leg swelling that eventually progressed to catastrophic antiphospholipid syndrome. She developed pulmonary embolisms, axillary hematoma, and bilateral lower extremity digital gangrene. The treatment course consisted of anticoagulation, steroids, intravenous immunoglobulin (IVIG), above-knee amputation, and eventually rituximab. Diagnosis and treatment of digital ischemia can be complex, especially, in the setting of lupus where the differential diagnosis is broad. A high index of suspicion for CAPS is essential for early diagnosis and treatment.

## Introduction

Catastrophic antiphospholipid syndrome (CAPS) is a life-threatening variant of antiphospholipid syndrome (APS), and it is distinguished on the APS spectrum by its increased intensity and extent of thrombotic outcomes. Diagnostic criteria for definitive CAPS involve multi-organ involvement, manifestation of symptoms within one week, histological proof of thrombosis, and antiphospholipid antibodies positivity. Please see Table 1 for complete diagnostic criteria. Less than 1% of patients with APS develop CAPS, and the demographic of patients affected is primarily females, 37 ± 14 years old, and have underlying primary APS or systemic lupus erythematosus (SLE) [1]. There are multiple thoughts on what triggers the progression from APS to CAPS. The inciting factors include systemic inflammatory response syndrome (SIRS)-provoking processes such as infections, surgeries, or malignancy. When there is fulminant CAPS, first-line treatment includes anticoagulation, steroids, plasmapheresis and/or intravenous immunoglobulin (IVIG). There are some reported cases of refractory CAPS, which is defined by either the patient dying or developing recurrent CAPS despite first-line therapy. Currently, in the literature, there are only anecdotal reports of three biologics used in refractory CAPS: rituximab, defibrotide, and eculizumab. The patient in the following case report is an example of someone who did not respond to first-line treatment of CAPS and was subsequently started on rituximab.

## Case presentation

**Figure 1:**
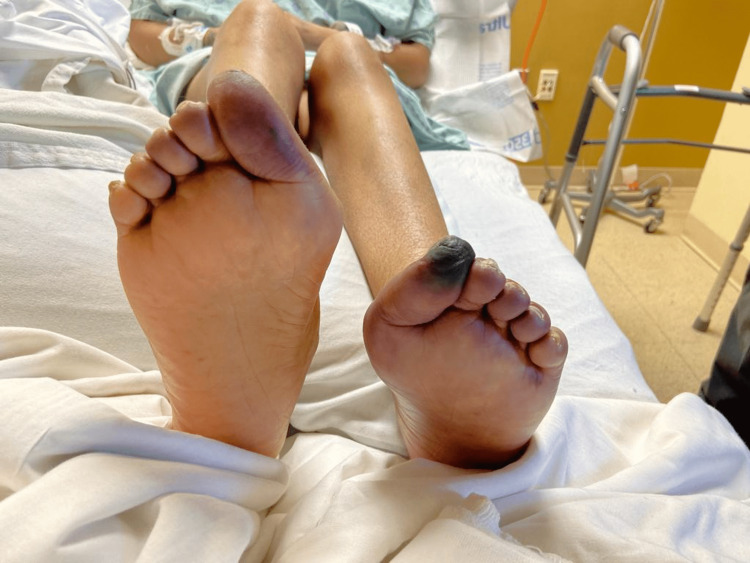
Earliest documentation of left toe ischemia

A 31-year-old female with lupus and end-stage renal disease secondary to lupus nephritis (on peritoneal dialysis) presented to the emergency room for chest pain, shortness of breath, and bilateral leg swelling with bluish discoloration of the left big toe (Figure 1).

She was diagnosed with lupus in 2010 with the initial symptoms being pleuritic chest pain and arthralgia. She was started on prednisone 10mg daily and hydroxychloroquine 200mg twice daily. By 2016, laboratory results and renal biopsies showed the patient had lupus nephritis stage III. The home regimen expanded to include mycophenolate mofetil 1gm daily and calcium with vitamin D. However two years later, it was discovered that the patient has not been taking her medications except for prednisone. She stated the amount of medications made compliance difficult. By 2022, the patient developed end-stage renal disease and was started on peritoneal dialysis. Unfortunately, the patient continued not to be compliant with either nightly home peritoneal dialysis or lupus medications.

Workup in the emergency department consisted of a CT chest which revealed a pulmonary embolism (PE) in the left upper segmental arteries. A heparin drip was subsequently started. The echocardiogram was negative for any wall motion abnormalities, pericardial effusions, or abnormal filling pressures. Laboratory reports revealed C-reactive protein (CRP) 423.6mg/L (normal <5mg/L), erythrocyte sedimentation rate (ESR) 87mm/hr (normal <20mm/hr), C3 107mg/dL (normal 87-200mg/L), C4 9mg/dL (normal 19-52mg/L), DNA immunoglobulin (Ig)G titer 1:160, Lupus anticoagulant positive, anticardiolipin (aCL) IgG antibody negative, aCL IgM antibody 15.3 (<15 MGL), anti-Beta 2 Glycoprotein (aβ2GPI) IgA 10.42 (1-20 SAU), aβ2GPI IgG 4.39 (1-20 SGU), and aβ2GPI IgM 4.83 (1-20 SMU). CT angiogram of the lower extremity showed no proximal vessel occlusion.

**Figure 2:**
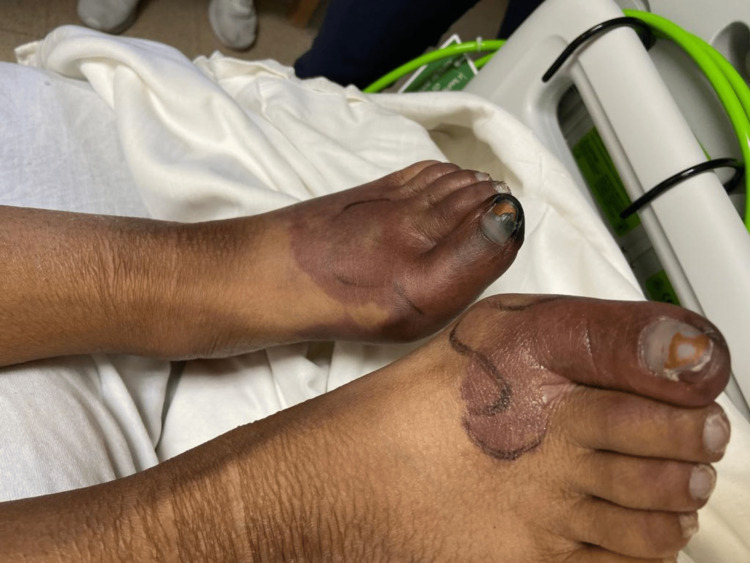
Progression of ischemic changes in both feet Progression of ischemic changes in left foot beyond previously demarcated lines with increased involvement of right toe

On initial physical exam, there was notable tender hyperpigmentation on the left foot as well as tender superficial vasculature on the right foot. This made SLE vasculitis with thrombophlebitis the initial suspicion. The patient received pulse dose steroids (250mg IV methylprednisolone for five days) followed by prednisone 60mg daily. Severe Raynaud’s phenomenon with gangrene was also concurrently suspected. The patient was given amlodipine, sildenafil, and topical nitrates to optimize blood flow to the feet. She also received hyperbaric oxygen therapy for several days, but it was held since it made her feel cold. Despite these efforts, the right foot progressed to develop a red hue and the hyperpigmentation in the left foot progressed beyond previously marked lines of demarcation (Figure 2).

**Figure 3:**
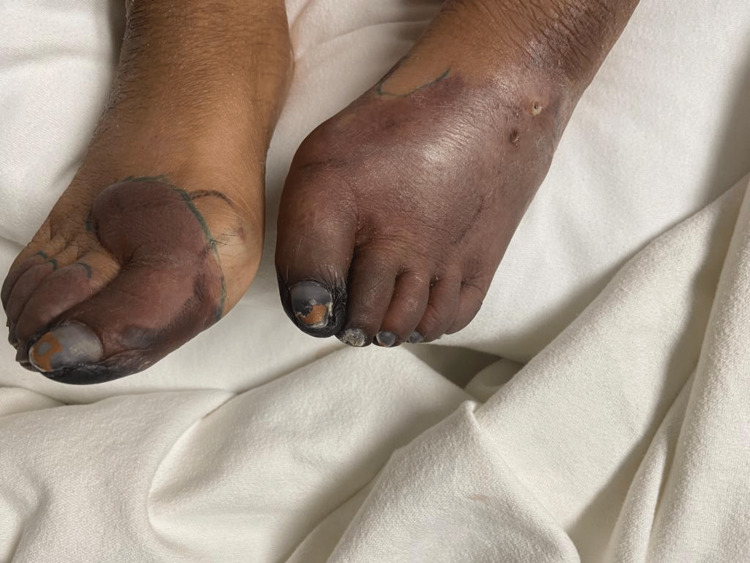
Progression of ischemia into nailbeds

**Figure 4:**
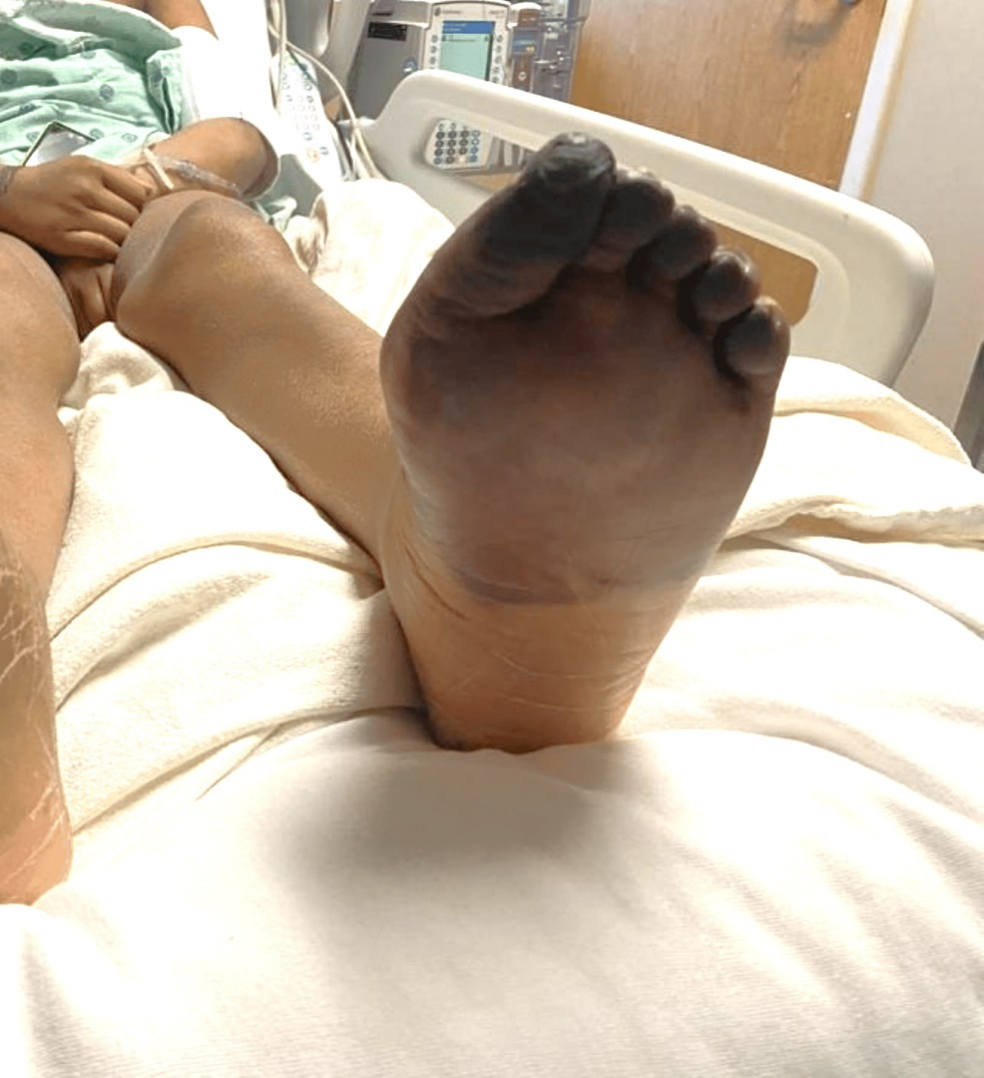
Ischemia progresses on the plantar surface

Therapy was escalated to IV epoprostenol for resistant Raynaud's syndrome, but despite efforts, black, ischemic changes extended into the nailbeds bilaterally and progressed even further on the plantar surface (Figures 3, 4). 

A 3mm punch biopsy of the left dorsal foot was done to differentiate between vasculitis and vasculopathy. Results showed fibrin thrombi occluding the vessels and papillary dermis, consistent with vasculopathy. With histological proof of thrombosis despite aggressive treatment, calciphylaxis, and probable CAPS became the leading diagnoses. Nephrology had been following throughout the course and therapy for possible calciphylaxis had already been concurrently running. She received sodium thiosulfate at the end of each hemodialysis session on top of daily cinacalcet and phosphate binders.

Throughout this time, not only did ischemic changes progress to involve most of her left foot, but the patient developed a right axillary hematoma and a new PE in the right lower segment of the lung. Incision and drainage of the hematoma were performed and 350mL of the hematoma was drained and another 400-500mL of a “persistent, organized clot” was digitally dissected. Now that the axilla is involved, the patient has met the criteria for definite CAPS with the criteria being the following: thrombosis affecting lungs, feet, and right axilla (Criterion One), manifestations of these clots are developed simultaneously (Criterion Two), histological proof of microvascular occlusion demonstrated from the forefoot (Criterion Three), and patient is positive for lupus anticoagulant, aβ2GPI, and aCL (Criterion Four) (Table 1).

**Table 1:** Diagnostic criteria for CAPS CAPS: Catastrophic antiphospholipid syndrome; aPL: Antiphospholipid antibody Reference [1]

Criteria
Involvement of 3+ organs/tissues
Manifestations developing simultaneously or within 1 week
Histological evidence of intravascular occlusion
Presence of antiphospholipid antibodies (lupus anticoagulant, anticardiolipin antibodies, and/or anti-beta2-glycoprotein I antibodies)
Definite CAPS:
1-4
Probable CAPS:
All 4 criteria, except for only sites of tissue involvement or
All 4 criteria, except for the laboratory confirmation at least 6 weeks apart due to the early death of a patient never tested for aPL before the catastrophic APS or
Criteria 1, 2, and 4 above or
1, 3, and 4 and the development of a third event in more than a week but less than a month, despite anticoagulation

**Figure 5:**
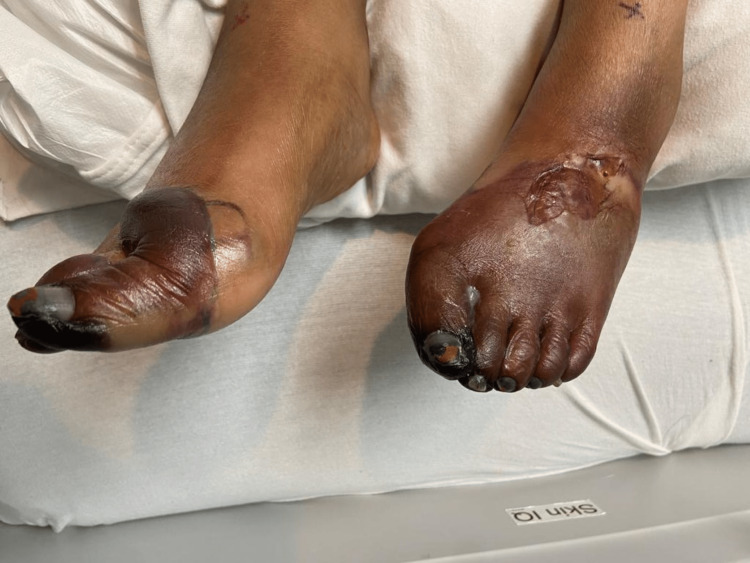
Gangrenous toes with blistering changes from edema

**Figure 6:**
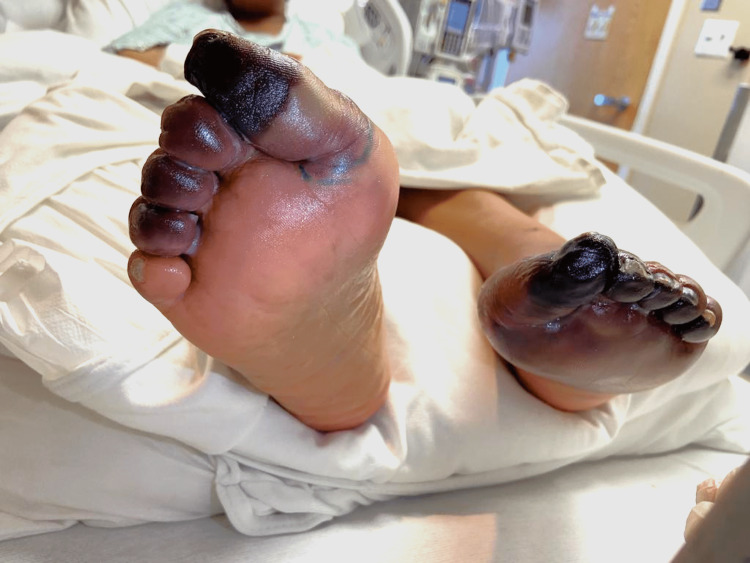
Gangrene from plantar surface

Five-day therapy of IVIG was started immediately. Platelets that once dropped from a baseline of >300 k/mm to a minimum of 79 k/mm slowly increased to a peak of 160 k/mm. Demarcation of ischemia of the foot has also ceased to expand. However, this positive response to therapy only lasted a couple of days before platelet count started to downtrend again and skin findings demonstrated progression from cyanosis to gangrene (Figures 5, 6). 

Ultimately, an above-knee amputation of the left lower extremity and transmetatarsal amputation of the right foot were performed. Biopsy of the vasculature from the amputation unsurprisingly revealed extensive atherosclerosis of the popliteal artery showing 60-70% occlusion and the anterior and posterior tibial arteries showing 90% occlusion. Due to the aggressive disease process that was resistant to first-line therapy, the patient was started on rituximab 1000mg.

## Discussion

Due to the rarity of this disease, an international registry of CAPS patients was formed in 2000 called “CAPS Registry” to aggregate information on this disease process. As of 2012, the registry revealed 280 patients, 72% females and 28% males, with a mean age of 37.14 years. The first clinical manifestation of a catastrophic episode is a pulmonary manifestation in 24% of cases, neurological feature in 18%, and renal failure in 18%. Meanwhile, during a catastrophic episode, intraabdominal involvement was seen in most, namely renal (71%), hepatic (33%), gastrointestinal (25%), and adrenal (13%). Also commonly seen are pulmonary complications (64%) namely acute respiratory distress syndrome and PE, cerebrovascular accidents, and skin complications (50%) namely digital gangrene, necrotic lesions, and multiple ecchymosis. The registry also revealed that the most common laboratory value seen in CAPS is thrombocytopenia (46%) with platelets less than 100 k/mm. Prevalent hypercoagulable antibodies were lupus anticoagulant (82%), IgG isotype of anticardiolipin antibody (aCL) (83%), and IgM aCL isotype(38%) [2].

From the CAPS Registry, there is a clear delineation between APS and CAPS in the way vasculature is affected. APS primarily affects macrovasculature whereas CAPS manifests as microvascular thrombosis. There are several precipitating factors for CAPS observed in 50% of CAPS cases which include surgery, withdrawal of anticoagulation, obstetric complications, neoplasia, and concomitant SLE flare [3]. 

More recently discovered precipitants that aligned with our patient’s presentation are thrombocytopenia (seen in 20-30% of APS cases versus 65% of CAPS cases), elevated levels of ferritin, and possibly low levels of vitamin D [4]. Thrombocytopenia may reflect widespread thrombosis which given the nature of ischemia, a cascade of cytokine and chemokine expression follows and can cause more clots and disruption to the fibrinolysis-thrombogenesis process. Therefore, vascular occlusion can precipitate the progression of CAPS. Besides being an iron storage protein, ferritin is an acute-phase reactant. Agmon-Levin et al. studied ferritin and its association with APS and CAPS and found that hyperferritinemia was present in 71% of CAPS patients with levels > 1000ng/mL (p<0.001) [5]. Ferritin enhances the expression of intracellular cell adhesion molecule 1 (CAM1), which is known to be involved in APS-related thrombosis [5]. Therefore, rather than reflecting an inflammatory state, ferritin may also be pathogenic in CAPS by regulating proinflammatory molecules. Vitamin D has immunoregulatory properties, such as differentiation from monocyte to macrophage, preventing them from releasing cytokines and chemokines, as well as regulatory effects on interleukin (IL)-12, IL-10, and B cells [4]. In reference to our patient, her platelets dropped to a low of 79 k/mm, ferritin level was elevated at 1,427 ng/mL and vitamin D was significantly low at < 7 ng/mL.

According to a study looking into the mortality of 250 patients with CAP, systemic lupus erythematosus (SLE) was found to be the most significant contributing factor to mortality, more so than primary APS (57% vs 39%, p<0.003) [6]. When it comes to treatment, it is imperative to consider preventative measures. In a patient at risk for CAPS, infections (regardless of how trivial) should be treated in a timely manner. When the patient undergoes surgery, anticoagulation should be running perioperatively. After childbirth, the patient should continue anticoagulation for a minimum of six weeks. In fulminant CAPS, there is a higher survival rate in those treated with the combination therapies of anticoagulants (ACs) plus corticosteroids (CS) plus plasmapheresis or ACs plus CS plus plasmapheresis and/or IVIG compared to those who were not (77% vs 55.4%, p=0.083) and (69% vs 54.4%, p=0.089). When the disease course is refractory to the aforementioned combination therapy, biologics come into consideration. Rituximab, defibrotide, and eculizumab are three biologics that are still relatively new in treating CAPS with only case reports to support efficacy. Rituximab demonstrated antiphospholipid antibody (aPL) resolution in three of nine CAPS patients [7].

Our case is consistent with what has been studied about CAPS in that our patient had thrombocytopenia, low vitamin D levels, and hyperferritinemia. These properties were recently reviewed as possible precipitants. Her concurrent SLE may also be a risk factor for her increased morbidity of the disease, as studied by Bucciarelli et al. [6]. The surprising component is that triple therapy with AC, CS, and IVIG did not have a significant effect on recovery for the patient. Per Espinosa et al., biologics can be used in those who have refractory disease to first-line therapy, although the results of using biologics are still very new and no controlled studies have been published.

## Conclusions

The diagnosis and treatment of digital ischemia can be complex, especially in the setting of lupus where the differential diagnosis is broad. Accelerated atherosclerotic disease, vasculitis, Raynaud's, and APS are all examples of differentials to consider. CAPS on the other hand is also difficult to diagnose, and a high index of suspicion is essential for early diagnosis. Our case presents a typical example of where multiple treatment modalities for all differential diagnoses had failed before CAPS was detected.

This study has one potential limitation. The patient's lack of response to first-line therapy for CAPS could be due to her noncompliance with lupus medications since early on in her diagnosis. Since SLE is observed to be the highest risk factor for mortality and morbidity in CAPS, having it be uncontrolled at baseline has potentially huge implications on patient outcomes.
